# Supplementary material for: Robust Bain distortion in the premartensite phase of a platinum-substituted Ni2MnGa magnetic shape memory alloy
Source: Nat Commun. 2017 Oct 18;8:1006. doi: 10.1038/s41467-017-00883-z (PMC5647333; doi:10.1038/s41467-017-00883-z)
Supplement: Supplementary file 1 — Supplementary Information [file 41467_2017_883_MOESM1_ESM.pdf]

Supplementary Table 1: Atomic positions (x,y,z) and amplitudes (A_i) of the modulation function, obtained from the Rietveld refinement using synchrotron x-ray powder diffraction data for the premartensite (PM) phases PM (T1) at 240 K and PM (T2) at 225 K. Parameters in red color are correspond to the PM (T2) phase. Here i =1 as only first order satellites are observed and B_i = 0 restricted by symmetry.

Crystal system		Orthorhombic		
Space group		*Immm(00γ)s00*		
Basic Cell (Å)	T= 240 K	*a**=4.13372(25),	*b**= 5.84160 (30),	*c**=4.13257(12)
	T= 225 K	*a**=4.13563(26),	*b**=5.82762(20),	*c**=4.13714(19)
Modulation vector	T= 240 K	**q**= 0.325 *c**		
	T= 225 K	**q**= 0.33761(5)*c**		

Atom	Wyckoff position	Modulation amplitude	*x*	y	z	*Occ.*	$U_{iso}(Å^2)$
Ni	4*h*		0.5	0.25	0	1.9	0.0115(19) [0.0122(6)]
Pt	4*h*		0.5	0.25	0	0.1	0.0115(19) [0.0122(6)]
		A_1	0.017(18) [0.0194(18)]	0	0		
Mn	2*a*		0	0	0	1	0.005(2) [0.0065(10)]
		A_1	0.03(2) [0.017(7)]	0	0		
Ga	2*d*		0	0.5	0	1	0.011(2) [0.0106(7)]
		A_1	0.020(17) [0.018(5)]	0	0		

Supplementary Table 2: Lattice parameters, space group and atomic positions of 3D rational approximate structure of $Ni_{1.9}Pt_{0.1}MnGa$ derived from (3+1) D incommensurate PM (T1) at 240 K and PM (T2) at 225 K.

Crystal system: Orthorhombic
Space Group: *Pnmn*
Lattice parameters:
T= 240 K: a= 4.13373 Å, b= 5.83968 Å, c= 12.40066 Å
T= 225 K: a= 4.138969 Å, b= 827392 Å, c= 12.40434 Å

Atoms	Wyckoff position	x	x [225 K]	y	z	Occ.	$U_{iso}(Å^2)$	$U_{iso}(Å^2)$ [225 K]
Mn1	2b	0	0	0	0	1	0.004796	0.006459
Mn2	4g	0.0244	0.0146	0	1/3	1	0.004796	0.006459
Ga1	2a	0	0	1/2	0	1	0.011083	0.01063
Ga2	4g	0.0176	0.0152	1/2	1/3	1	0.011083	0.01063
Ni1	4f	1/2	½	1/4	0	0.95	0.011497	0.012245
Ni2	8h	0.5144	0.5168	1/4	1/3	0.95	0.011497	0.012245
Pt1	4f	1/2	½	1/4	0	0.05	0.011497	0.012245
Pt2	8h	0.5144	0.5168	1/4	1/3	0.05	0.011497	0.012245

Supplementary discussion. In supplementary Table 1 we compared the structure of PM (T1) at 240 K and PM (T2) at 225 K obtained from the Rietveld refinements using (3+1) D super-space group analysis. Both the structures have similar symmetry and basic structure but the amplitude of modulation changes, which is equivalent to the displacement of atomic position. We have also compared the structural parameters for 3D rational approximant in supplementary Table 2, which shows a clear change in the atomic position (x component) between premartensite (PM) phases PM (T1) and PM (T2).

Supplementary Methods:

To investigate the modulated structure of two premartensite (T1 and T2) and martensite phases in $Ni_{1.9}Pt_{0.1}MnGa$ super-space (3+1) D formalism was used[1-4]. In (3+1)D super-space group approach, the deviation of the atoms from their average atomic positions x_i (i = 1 to 3)

in the basic structure is accounted by the atomic modulation functions $u\ (\overline{x}_4)$ along the x_4 axis of the super-space in terms of superposition of harmonic waves. The atomic positions (x_i) of the modulated phase therefore can be given as:

$$x_i = \overline{x_i} + u^j(\overline{x}_4) \qquad (1)$$

$$u^j(\overline{x}_4) = \sum_{n=1}^{\infty}[A_n^j \sin(2\pi n\overline{x_4}) + B_n^j \cos(2\pi n\overline{x_4})] \qquad (2)$$

where $\overline{x_i}$ is the general atomic position in the basic structure, n is the order of the Fourier series, which is equivalent to the order of the satellite reflection, A_n^j and B_n^j are the Fourier amplitudes of the displacement modulation of the jth atom.

Supplementary References:

1 Dusek, M., Petricek, V., Wunschel, M., Dinnebier, R. E. & van Smaalen, S. Refinement of modulated structures against X-ray powder diffraction data with JANA2000. *Journal of Applied Crystallography* **34**, 398-404, doi:doi:10.1107/S0021889801003302 (2001).

2 Janssen, T., Janner, A., Looijenga-Vos, A. & De Wolff, P. International Tables for Crystallography, Vol. C. *Mathematical, Physical and Chemical Tables* (1992).

3 De Wolff, P. The pseudo-symmetry of modulated crystal structures. *Acta Crystallographica Section A: Crystal Physics, Diffraction, Theoretical and General Crystallography* **30**, 777-785 (1974).

4 Van Smaalen, S. *Incommensurate crystallography*. (Oxford University Press, USA, 2007).
